# A case of polyarteritis nodosa limited to the right calf muscles, fascia, and skin: a case report

**DOI:** 10.1186/1752-1947-5-450

**Published:** 2011-09-12

**Authors:** Saad Ahmed, Joanne Kitchen, Samuel Hamilton, Francesca Brett, David Kane

**Affiliations:** 1 Department of Rheumatology, The Adelaide and the Meath Hospital incorporating the National Children's Hospital, Dublin, Ireland; 2 Radiology Department, The Adelaide and the Meath Hospital incorporating the National Children's Hospital, Dublin, Ireland; 3 Department of Histopathology, Beaumont Hospital, Dublin, Ireland

## Abstract

**Introduction:**

Limited polyarteritis nodosa is a rare benign disease that usually responds well to systemic corticosteroid treatment. We report a case limited to calf muscles, fascia, and skin treated with local corticosteroid therapy directed to the affected areas by ultrasound guidance.

**Case presentation:**

A 36-year-old Caucasian woman presented with a 10-month history of progressive right calf pain and swelling, which were unresponsive to treatment with non-steroidal anti-inflammatory drugs and physiotherapy. An examination revealed a swollen tender right calf with indurated overlying skin. Laboratory investigations showed an erythrocyte sedimentation rate of 24 mm/hour and a C-reactive protein of 15 mg/dl. Full blood count, renal profile, and creatinine kinase level were normal. A full autoantibody screen and hepatitis B and C serology results were negative. A chest X-ray was unremarkable. Magnetic resonance imaging of the right leg revealed increased signal intensity in T2-weighted images and this was suggestive of extensive inflammatory changes of the gastrocnemius muscle and, to a lesser extent, the soleus muscle. There were marked inflammatory changes throughout the gastrocnemius muscle and the subcutaneous tissue circumferentially around the right lower leg. A biopsy of affected skin, muscle, and fascia showed histopathological features consistent with polyarteritis nodosa, including small-vessel vasculitis with fibrinoid changes in the vessel wall and intense perivascular and focal mural chronic inflammatory changes. Our patient declined treatment with oral steroids. She received a course of ultrasound-guided injections of steroid (Depo-Medrone, methylprednisolone) in the involved muscle area and commenced maintenance azathioprine with a good response.

**Conclusions:**

Limited polyarteritis nodosa is rare and affects middle-aged individuals. In most cases, treatment with moderate- to high-dose corticosteroids gives symptomatic relief within one week. Resistant cases require treatment with cytotoxics or intravenous immunoglobulins. This case demonstrates response to local targeted steroid therapy as an alternative to systemic steroids.

## Introduction

Classic polyarteritis nodosa is a multi-system, necrotizing vasculitis of small- and medium-sized muscular arteries in which involvement of the renal and visceral arteries is characteristic [1]. Limited forms of polyarteritis nodosa have been described, and the skin is the most common organ to be involved [2]. Cases of polyarteritis nodosa limited to gall bladder [3], pancreas [3], female [4] and male [5] genital tracts, kidneys [6], and gastrointestinal tract [7] have also been reported. Interest in these forms is based on their prognosis, which, in general, is more benign, and their quick response to corticosteroids alone [2]. Polyarteritis nodosa limited to calf muscles is very rare and only 14 case reports have been published. It commonly affects middle-aged individuals (average age of 40 years), and there is no significant sex variation [1]. Laboratory markers of inflammation (erythrocyte sedimentation rate and C-reactive protein) were elevated in all previous reports. Creatinine kinase is usually within normal limits. Only two reported cases had positive autoantibodies: a positive perinuclear anti-neutrophil cytoplasmic antibody in one [8] and a positive anti-phospholipid antibody in the other [9]. Unlike classic polyarteritis nodosa, which usually requires a combination of steroids and a cytotoxic drug such as cyclophosphamide for treatment [1], limited polyarteritis nodosa usually responds well to treatment with corticosteroids alone with symptomatic relief within one week in most cases [10,11]. The dose of steroids used varied between 15 and 60 mg of prednisolone for initial treatment and 5 and 30 mg for maintenance. Two cases were reported to be resistant to corticosteroids but both of them responded well to intravenous immunoglobulin treatment and symptomatic response was rapid; however, one of the cases relapsed after six months and needed an increase in the oral steroid dose and the addition of methotrexate [10]. Polyarteritis nodosa limited to calf muscles, fascia, and skin is a rare disease that runs a benign course and usually responds well to corticosteroid treatment. Resistant cases can be treated with cytotoxics such as azathioprine and methotrexate. The use of intravenous immunoglobulins is reported to induce a rapid symptomatic recovery in resistant cases, which may require cytotoxics for maintenance. The risk of progression to systemic disease is low, but close long-term follow-up of these patients may be advisable [12].

## Case presentation

**Figure 1:**
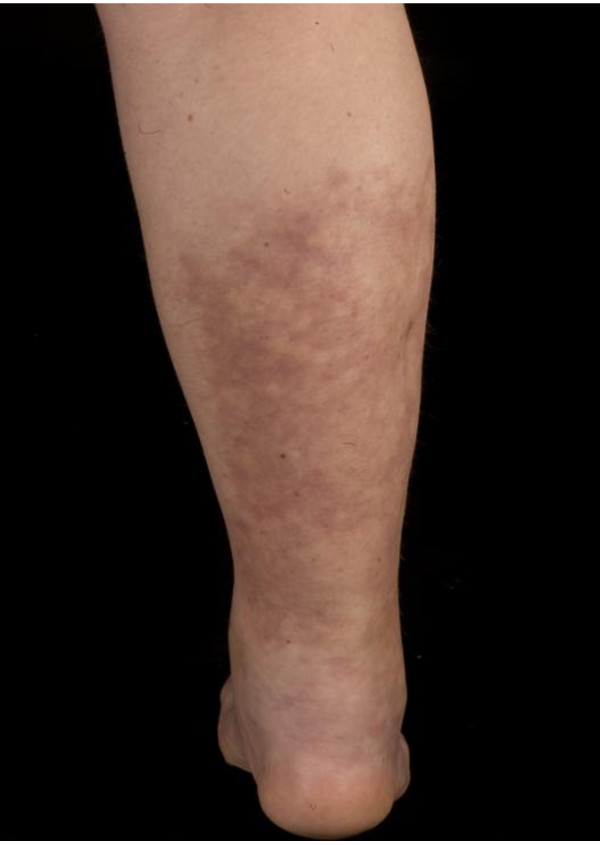
**Right calf with swelling and induration of skin at presentation**.

**Figure 2:**
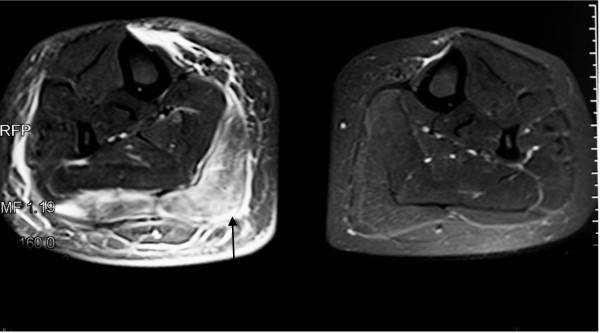
**Magnetic resonance imaging images of both legs**. Increased signal intensity in the T2-weighted image of the right gastrocnemius muscle and subcutaneous tissue (arrow) was caused by severe inflammatory changes, whereas the left leg is normal.

**Figure 3:**
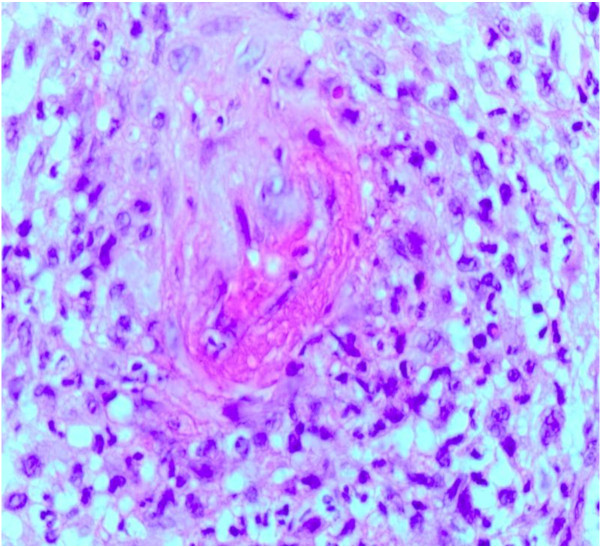
**Fibrinoid necrosis of the vessel wall with surrounding perivascular lymphocytic infiltrates**. Stain: hematoxylin and eosin. Magnification: ×40.

A 36-year-old Caucasian woman presented with a 10-month history of progressive right calf pain and swelling that severely limited walking and standing. Her condition had been diagnosed as Achilles tendinitis but had not responded to treatment with non-steroidal anti-inflammatory drugs and physiotherapy. On examination, her right calf was swollen and tender with induration and thickening of overlying skin (Figure 1). In laboratory investigations, there was an elevated acute-phase response (erythrocyte sedimentation rate of 24 mm/hour and C-reactive protein of 15 mg/dl). Full blood count and levels of creatinine kinase, urea, and electrolytes were normal. Levels of anti-nuclear antibodies, extractable nuclear antigens, and anti-cytoplasmic antibodies were negative. The results of hepatitis B and C serologies were negative. A chest X-ray was unremarkable. Magnetic resonance imaging of the right leg revealed increased signal intensity in T2-weighted images and this was suggestive of extensive inflammatory changes of the gastrocnemius muscle and, to a lesser extent, the soleus muscle. Inflammatory changes of the subcutaneous tissues in the right lower leg were also found (Figure 2). Our patient underwent a biopsy of the involved skin, fascia, and muscles. Histopathology revealed mixed perivascular and interstitial inflammatory infiltrate predominantly of lymphocytes, plasma cells, and occasional eosinophils involving the deep fascial tissue, whereas the skin and subcutaneous fat were relatively unremarkable. Cutaneous changes could be secondary to tissue remodeling and edema due to the subjacent inflammatory process. In one fragment of muscle, there was small-vessel vasculitis with fibrinoid changes in the vessel wall and intense perivascular and focal mural chronic inflammatory changes. No granuloma was seen (Figure 3). Histopathological features were consistent with polyarteritis nodosa. Our patient declined treatment with oral steroids. She received four courses of ultrasound-guided injections of steroid (80 mg of Depo-Medrone, methylprednisolone) in the involved muscle and subcutaneous tissues at two weekly intervals. Azathioprine 50 mg once daily was added. After three months, our patient responded to treatment and no longer required analgesia when walking. A softening of the localized rash was also observed. Follow-up magnetic resonance imaging at five months revealed almost complete resolution of edema. Our patient showed no signs of recurrence or progression to systemic polyarteritis nodosa more than one year after finishing treatment.

## Conclusions

We describe the first case using localized corticosteroid therapy to treat polyarteritis nodosa limited to muscles, fascia, and skin, thus minimizing potential complications of systemic corticosteroid use.

## Consent

Written informed consent was obtained from the patient for publication of this case report and any accompanying images. A copy of the written consent is available for review by the Editor-in-Chief of this journal.

## Competing interests

The authors declare that they have no competing interests.

## Authors' contributions

SA, the main author, did the literature review and wrote the paper and submitted it to the journal. JK wrote the Abstract and assisted in writing the paper. SH offered the magnetic resonance imaging image and wrote the description of the radiological changes. FB performed the histological examination of the calf biopsy and wrote the histological section. DK did the ultrasound-guided injections of steroid, was the consultant who treated the patient, and was a major contributor in writing the paper. All authors read and approved the final manuscript.
